# Epitope mapping and kinetics of CD4 T cell immunity to pneumonia virus of mice in the C57BL/6 strain

**DOI:** 10.1038/s41598-017-03042-y

**Published:** 2017-06-14

**Authors:** Lana Vandersarren, Cedric Bosteels, Manon Vanheerswynghels, James J. Moon, Andrew J. Easton, Gert Van Isterdael, Sophie Janssens, Bart N. Lambrecht, Mary J. van Helden

**Affiliations:** 1 Laboratory of Immunoregulation and Mucosal Immunology, VIB Center for Inflammation Research, Ghent, Belgium; 2 Department of Internal Medicine, Ghent University, Ghent, Belgium; 3 GROUP-ID Consortium, Ghent University and University Hospital, Ghent, Belgium; 4 Department of Biomedical Molecular Biology, Ghent University, Ghent, Belgium; 5 Center for Immunology and Inflammatory Diseases, and Division of Pulmonary and Critical Care Medicine, Massachusetts General Hospital; and Harvard Medical School, Boston, MA 02114 USA; 6 School of Life Science, University of Warwick, Coventry, United Kingdom

## Abstract

Pneumonia virus of mice (PVM) infection has been widely used as a rodent model to study the closely related human respiratory syncytial virus (hRSV). While T cells are indispensable for viral clearance, they also contribute to immunopathology. To gain more insight into mechanistic details, novel tools are needed that allow to study virus-specific T cells in C57BL/6 mice as the majority of transgenic mice are only available on this background. While PVM-specific CD8 T cell epitopes were recently described, so far no PVM-specific CD4 T cell epitopes have been identified within the C57BL/6 strain. Therefore, we set out to map H2-IA^b^-restricted epitopes along the PVM proteome. By means of *in silico* prediction and subsequent functional validation, we were able to identify a MHCII-restricted CD4 T cell epitope, corresponding to amino acids 37–47 in the PVM matrix protein (M_37–47_). Using this newly identified MHCII-restricted M_37–47_ epitope and a previously described MHCI-restricted N_339–347_ epitope, we generated peptide-loaded MHCII and MHCI tetramers and characterized the dynamics of virus-specific CD4 and CD8 T cell responses *in vivo*. The findings of this study can provide a basis for detailed investigation of T cell-mediated immune responses to PVM in a variety of genetically modified C57BL/6 mice.

## Introduction

Pneumoviruses were formerly assigned to the paramyxoviral subfamily *Pneumovirinae*, but following a recent taxonomy update this subfamily was elevated to family status (the family of *Pneumoviridae*), and the genus Pneumovirus was renamed to Orthopneumovirus^1^. Members of the *Pneumoviridae*, are enveloped viruses with negative-sense non-segmented RNA genomes. Closely related Orthopneumoviruses, such as human respiratory syncytial virus (hRSV), bovine respiratory syncytial virus (bRSV) and pneumonia virus of mice (PVM) are highly species-specific and can cause severe respiratory infections in their natural hosts^2^. Among these, hRSV is one of the leading causes of airway morbidity and infant hospitalization worldwide. Therefore, modeling hRSV disease *in vivo* is a crucial first step in the further understanding of antiviral immune responses and the development of novel therapies and preventive measures against RSV pathogenesis. So far, most studies have relied on the use of hRSV itself to infect inbred laboratory mouse strains, particularly BALB/c, to unravel different aspects of RSV pathology^3, 4^. However, as pneumoviruses display a narrow host range, human RSV does not replicate robustly in murine tissue and inadequately reproduces specific features of human RSV disease in mice^2, 4, 5^. More recently, infection of mice with PVM, the natural rodent-specific variant of hRSV, has been proposed as an alternative experimental model for human RSV infection^6^. PVM and hRSV display marked genomic similarity, as every hRSV viral protein has a counterpart in PVM even though direct sequence homology is limited^7^. More importantly, the PVM infection model accurately mimics many of the clinical and pathological hallmarks of RSV disease in human infants^2, 4^.

Even though RSV replicates poorly in mice, several groups have extensively studied many aspects of T cell biology using various approaches to induce hRSV-driven disease. Overall, these studies have revealed that T cells contribute to viral clearance, but are also the main drivers of immunopathology^8–12^. Because of this apparent dual role, T cell responses have also been evaluated in the PVM model. A comprehensive study by Frey and colleagues illustrated a key role for both CD4 and CD8 T cells in virus control and induction of PVM-mediated disease^13^. In the context of CD4 T cell responses, it was demonstrated that IL21R KO mice survive longer in response to PVM infection, suggesting that activated CD4 T cells, the main producers of IL21, may contribute to pathology^14^. Adoptive transfer studies and peptide-immunization studies have revealed that as well as their contribution to immunopathology during primary infection, T cells can also provide protection against severe PVM-induced disease^15, 16^. Overall, these studies suggest the existence of a tight balance between beneficial and detrimental effects caused by T cells during pneumovirus infection, however, underlying molecular mechanisms remain elusive.

The PVM infection model is well-accepted for studying severe RSV-induced disease, however insufficient tools are currently in place to study T cell responses in great detail. While hRSV- or PVM- specific T cell epitopes have been described particularly for BALB/c (H2^d^) mice, most transgenic and knockout mice are primarily available on a C57BL/6 (H2^b^) background^15, 17–19^. Recently, Walsh and colleagues identified PVM-specific H2^b^-restricted CD8 T cell epitopes in C57BL/6 mice^20^. However, so far, no PVM-specific CD4 T cell epitopes have been identified in the context of PVM-infected C57BL/6 mice. While T cell kinetics during pulmonary PVM infection have been described in response to PVM strain J3666 in BALB/c mice^16^ and PVM strain 15 in C57BL/6 mice^13^, to our knowledge a detailed kinetic documentation of both CD4 and CD8 T cell responses against PVM strain J3666 is currently lacking in C57BL/6 mice. Therefore, the aim of this study was to map CD4 T cell epitopes along the PVM proteome and determine the dynamics of the PVM-specific CD4 and CD8 T cell response following PVM infection in C57BL/6 mice.

## Results

### Clinical features of disease manifestation and T cell dynamics in response to PVM J3666 infection in C57BL/6 mice

**Figure 1:**
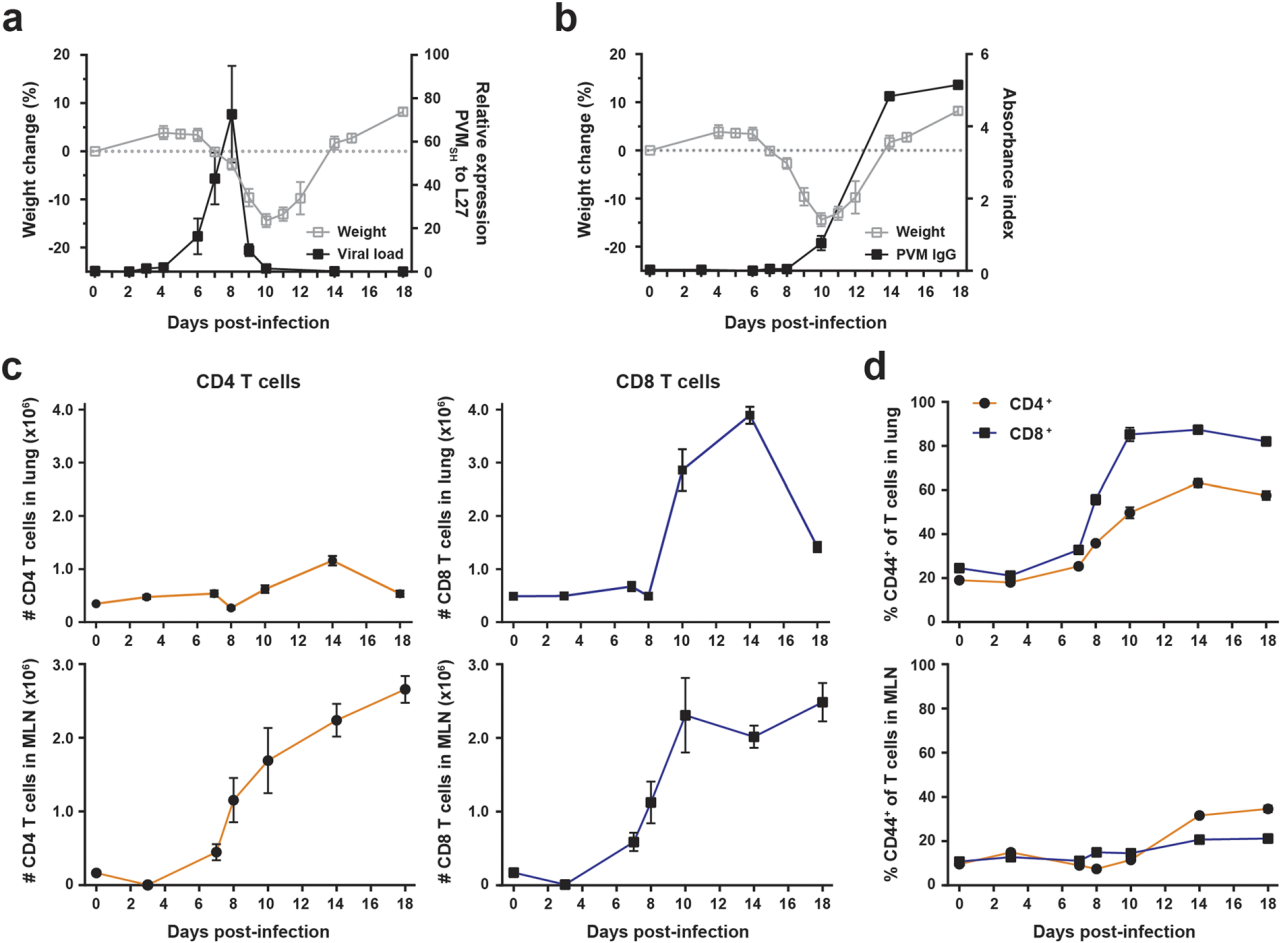
Weight change kinetics, viral load and dynamics of T cell responses in PVM-infected mice. 8 week old C57BL/6 females were infected i.t. with a sub-lethal dose of PVM strain J3666 and sacrificed at the indicated days post-infection. (**a**) Weight change and viral load kinetics. Open gray squares depict percentage difference in mean weight relative to day 0 as depicted by the dotted line (n = 5). Filled dots show expression levels of the PVM SH gene relative to L27 as determined by qRT-PCR on lung tissue (n = 3–9 per time point). (**b**) Percentage weight loss (described in a; open squares) and levels of PVM-specific antibodies in serum (filled squares; n = 4 per time point), represented as the absorbance index relative to a positive control. (**c**) Absolute numbers of CD4 (orange) and CD8 (blue) T cells in the lung and MLN, as determined by flow cytometry (n = 5 per time point). Gating strategy, see Supplementary Fig. S1. (**d**) Frequency of CD44^+^ T cells in lung and MLN as percentage of total CD4 (orange) or CD8 (blue) T cells (n = 5 per time point). All results are shown as mean ± SEM and are representative of two (**a** and **b**) or three (**c** and **d**) independent experiments. SH, small hydrophobic protein; L27, ribosomal protein L27; MLN, mediastinal lymph node.

Two well-characterized strains of PVM, strain 15 and strain J3666, are commonly used for research purposes^7, 21–23^. To investigate the T cell response during PVM disease, we administered a sub-lethal dose of PVM strain J3666 intratracheally (i.t) to C57BL/6 mice and weight loss was monitored as a clinical measure for disease **(**Fig. 1a **)**. Mice started to gradually lose body weight at day 7 post-infection (pi), with a maximal weight loss of approximately 15% around 10–11 days pi. All mice had recovered from disease around 14 days pi. Viral load was assessed by means of qRT-PCR on lung tissue and virus RNA was first detectable from day 6 pi onwards, with a peak around day 8 pi. This was followed by a phase of viral clearance and virus RNA was no longer detected by day 14 pi. The kinetics of weight loss and the viral load determined by qRT-PCR was the same as that described by Frey *et al*. using an infectious virus assay in the analysis of the role of T cells in PVM disease, with the peak of viral load seen at day 8 pi and the peak of PVM-induced weight loss being most pronounced during the phase of virus elimination **(**Fig. 1a **)**. Finally, PVM-specific antibodies in the serum were detectable at day 10 pi and reached maximal titers around 14 days pi, when mice had recovered from disease **(**Fig. 1a–b **)**.

We used this model to explore the dynamics of the CD4 and CD8 T cell responses upon primary infection, both at the site of infection (i.e. lung) and the draining mediastinal lymph node (MLN). The gating strategy used to define CD4 and CD8 T cell populations is depicted in Supplementary Fig. S1. The influx of both CD4 and CD8 T cells in the lung occurred between day 10 and 14 pi, albeit higher numbers of CD8 T cells were detected **(**Fig. 1c **, upper panels)**. A similar course was observed for total numbers of CD4 and CD8 T cells in the bronchoalveolar lavage (BAL) **(**Supplementary Fig. S1). In the MLN, expansion of the CD4 and CD8 T cell populations started around day 7–8 pi and numbers were still rising at day 18 pi **(**Fig. 1c **, lower panels)**. Recruitment of activated effector T cells within lung infiltrates was determined by CD44 expression^24^. The relative proportions of both CD44^+^ effector CD4 and CD8 T cells in the airways of PVM-infected mice started to increase at day 8 pi. From day 14 pi onwards, approximately 85% of CD8 T cells and 60% of CD4 T cells in the lung expressed high levels of CD44. In contrast, the relative proportions of CD44^+^ effector T cells in the MLN did not change substantially during the course of infection **(**Fig. 1d and Supplementary Fig. S1). Thus, activated effector T cells were recruited to the airways of PVM-infected mice around day 8 and reached maximal levels between day 10 to 14 pi.

### In silico prediction of MHCII-restricted PVM epitopes

Having characterized the general T cell responses upon PVM infection, we next aimed to study virus-specific T cell dynamics. Walsh and colleagues recently identified MHCI-restricted T cell epitopes in PVM-infected C57BL/6 mice, however, MHCII-restricted T helper epitopes have not yet been described in the C57BL/6 context. To identify novel PVM-specific CD4 T cell epitopes, the amino acid sequence of the PVM strain J3666 proteome was screened for peptides with the potential for high MHCII-binding affinity using the Immune Epitope Database (IEDB) and analysis resource Consensus tool^25, 26^. From the output of the online algorithm, the 2 highest-ranked non-overlapping peptides were selected for each of the 12 viral PVM proteins. Based on these criteria, a set of 24 predicted peptide sequences was obtained, which was evenly distributed along the PVM genome as summarized in Table 1.

**Table 1:** Predicted MHCII-restricted PVM epitopes.

Peptide sequence	Protein^1^	Position		Percentile rank^2^
		Start	End	
CNLLRPFVQAAKFIH	NS1	57	71	15,14
QTLTHWFTKNIVFSS	NS1	77	91	18,73
MNKFTQTISKPATIL	NS2	5	19	4,62
LLIEKFQPSLQNITR	NS2	49	63	6,43
IIGSYKGAPRNRELF	NP	333	347	9,68
VEGLFSGLFMNAYGA	NP	239	253	10,75
TIMVATAGPTTARDE	P	198	212	1
LRSSFKLPSPRVAAN	P	51	65	1,52
LLQILLNPLLPLPLH	P2	22	36	5
LTIRNTARSHAAQMI	P2	64	78	6,87
PKNMLYTVPSITPTN	M	122	136	1,89
TVWIPMFQTSLPKNS*	M	33	47	4,95
CTVHPNHPPPSYGVN	SH	64	78	4,4
QKLSFNKPQARQLYP	SH	98	112	8,59
MGRNFEVSGSITNLN	G	1	15	2,16
TIPRFTKPPTKTATH	G	94	108	4,44
LVIFNTKPIHPNTLT	F	11	25	5,18
ISTSKTYVSTAVLTT	F	358	372	6,99
KYSHKYWEWPLKTLM	M2-1	21	35	11,73
NRIYRFLDTNTDAMS	M2-1	43	57	15,75
NLTYDGSGPSTIIDA	M2-2	44	58	0,65
MIRLPKYYPAILHKM	M2-2	22	36	7,14
QHMFLPNHITPAQYI	L	1378	1392	2,02
VPMQFGGADPNVIYR	L	926	940	2,54

^1^PVM proteins: Non-structural protein (NS1 and 2), Nucleoprotein (NP), Phosphoprotein (P, P2), Matrix protein (M, M2-1, M2-2), Small hydrophobic protein (SH), Attachment glycoprotein (G), Fusion protein (F), RNA polymerase (L).

^2^The MHCII binding predictions were performed using the IEDB analysis source Consensus tool. The lower the percentile rank, the better the binders.

^*^Shows 100% homology to PVM strain 15; the underlined region delineates the core epitope M_37–47_, contained within the M_33–47_ sequence.

### Identification of a novel MHCII-restricted PVM epitope M_37–47_

**Figure 2:**
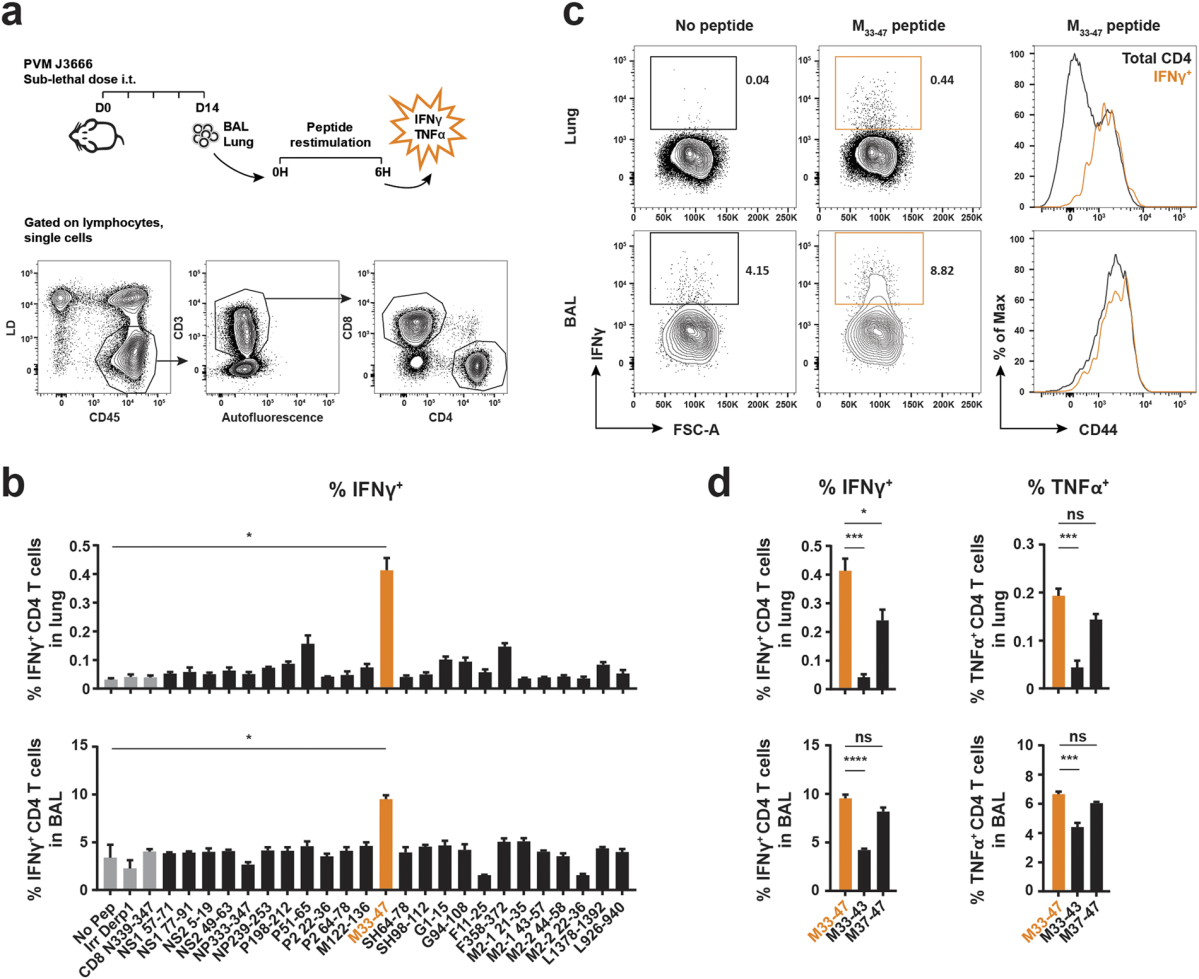
Identification of MHCII-restricted PVM epitopes recognized by CD4 T cells from PVM-infected mice. 8 week old C57BL/6 females were infected i.t. with a sub-lethal dose of PVM strain J3666 and sacrificed 14 days later. BAL and lung single-cell suspensions were restimulated for 6 h in the presence of Golgistop and PVM-specific peptides. T cells were evaluated for cytokine production by intracellular staining and flow cytometry analysis. (**a**) Schematic overview of the experimental setup and gating strategy to identify CD4 and CD8 T cell populations in BAL and Lung. (**b–d**) IFNγ or TNFα production by CD4 T cells, isolated from lung or BAL, after incubation with each of the predicted MHCII-restricted PVM peptides enlisted in Table 1. As negative controls, cells were incubated without peptide, with an irrelevant Derp1 CD4 peptide^27^, or with a MHCI-restricted PVM N_339–347_ peptide^20^ and are shown in gray. (**b**) IFNγ production by CD4 T cells in BAL and Lung. Data are depicted as frequency of IFNγ-producing cells among total CD4 T cells. (**c**) Left, representative FACS plots (gated on CD4^+^ cells) show the percentage of IFNγ^+^ CD4 T cells in response to restimulation with or without M_33–47_ peptide. Right, Histogram overlays depict CD44 expression levels of total CD4^+^ and gated IFNγ^+^ CD4^+^ populations (marked orange in left panel) following restimulation with M_33–47_ peptide. Data are normalized to and depicted as the percentage of the maximum count (% of max on the Y axis). (**d**) IFNγ and TNFα production by CD4 T cells following restimulation with M_33–47_, M_33–43_, or M_37–47_. Results are shown as mean ± SEM from three biological replicates. For each biological replicate 10 mice were pooled to obtain sufficient cell numbers for epitope screening. Data are representative of two independent experiments. For statistics, conditions restimulated with peptide were compared to the no-peptide control (**b**, Student’s *t* test with Welch correction) or the M_33–47_ peptide (**d**, ANOVA for multiple comparisons) as indicated. BAL, bronchoalveolar lavage; IFNγ, interferon gamma; TNFα, tumor necrosis factor alpha; MHCI/II, major histocompatibility complex class I or II; Derp1, *Dermatophagoides pteronyssinus* peptidase 1; Irr, irrelevant; ns, not significant.

Next, this panel of 24 predicted IA^b^-restricted PVM epitopes was validated for their capacity to stimulate CD4 or CD8 T cells isolated from PVM-infected mice. C57BL/6 mice were i.t. infected with a sub-lethal dose of PVM J3666 and sacrificed at 14 days pi, at the time of maximal CD4 T cell recruitment to the airways **(**Fig. 1c **)**. BAL and lung single-cell suspensions were restimulated *ex vivo* with each of the predicted MHCII-restricted PVM epitopes and cytokine production by T cells was evaluated by intracellular staining, using flow cytometry **(**Fig. 2a **)**. To determine background cytokine production, we also restimulated cells without peptide, with an irrelevant MHCII-restricted Derp1_128–149_ peptide^27^ or with a known MHCI-restricted N_339–347_ epitope^20^. Of the 24 peptides tested, only the M_33–47_ peptide potently induced IFNγ production above background in CD4 T cells isolated from both lung and BAL **(**Fig. 2b,c **)**. In the lungs, 0.3–0.5% of total pulmonary CD4 T cells responded to peptide M_33–47_, while in the BAL this was about 9–10% of total CD4 T cells, although background IFNγ levels were also higher in BAL compared to lung **(**Fig. 2b,c **)**. IFNγ^+^ CD4 T cells expressed high levels of CD44, consistent with an activated state **(**Fig. 2c **)**. We also assessed TNFα production by CD4 T cells and observed a similar cytokine pattern in response to the same panel of predicted peptides **(**Supplementary Fig. S2 **)**. Of note, CD8 T cells in the same cultures did not respond to any of the peptides summarized in Table 1, except to peptide NP_333–347_ **(**Supplementary Fig. S2), which included the GAPRNRELF sequence of the MHCI-restricted N_339–347_ epitope^20^.

For the development of MHCII tetramers, it was important to exclude that multiple binding registers existed within the M_33–47_ peptide sequence. To verify this, we used another IA^b^ peptide prediction algorithm^28^. Unexpectedly, we identified two overlapping epitopes within the M_33–47_ peptide sequence. In the context of MCHII tetramer design, it was essential to identify the core sequence of the M_33–47_ peptide. The reason for this is that the MHCII:peptide binding is flexible and anchorless. When a tetramer is generated using a peptide that contains overlapping epitopes, the MHCII molecules might display the peptide in different registers. Such a tetramer could thus consist of different epitope arms, which would reduce the effectiveness of the tetramer staining because of the loss of avidity. We therefore set-out to further identify the most potent core sequence within this immunodominant epitope. Cells isolated from PVM-infected mice were restimulated with two overlapping 11-mer peptides, M_33–43_ (TVWIPMFQTSL) and M_37–47_ (PMFQTSLPKNS), covering the initially identified 15-mer M_33–47_ sequence (TVWIPMFQTSLPKNS). Both in lung and BAL, only M_37–47_ elicited a robust IFNγ and TNFα response in CD4 T cells, albeit slightly less efficient than the full length M_33–47_, which most likely can be explained by the presence of multiple binding registers that activate a mixed pool of T cells **(**Fig. 2d **)**. Again, no significant cytokine production was detected in CD8 T cells from the same culture (data not shown). Overall, these data demonstrate that the M_37–47_ peptide is an IA^b^-restricted PVM epitope that is specifically recognized by CD4 T cells from PVM-infected C57BL/6 mice.

### Kinetics of PVM-specific CD4 and CD8 T cell responses in C57BL/6 mice

**Figure 3:**
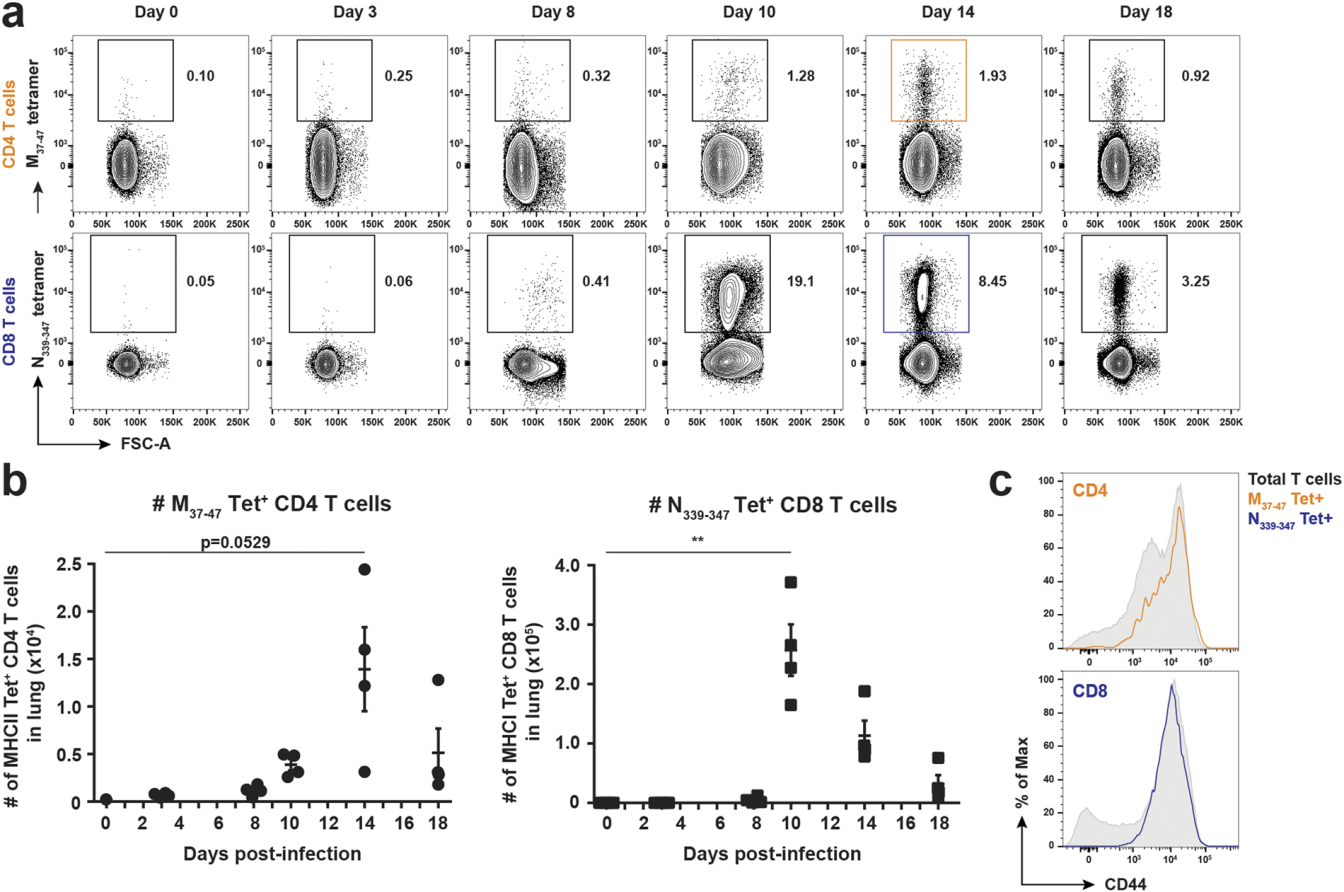
*In vivo* characterization of the virus-specific CD4 and CD8 T cell response in PVM-infected mice. 7 week old C57BL/6 females were infected i.t. with a sub-lethal dose of PVM strain J3666 or were used as uninfected controls (day 0). At the indicated time points post-infection, virus-specific CD4 and CD8 T cells in the lung were identified by flow cytometry, using M_37–47_ and N_339–347_ peptide-loaded MHC class II and MHC class I tetramers. (**a**) Representative FACS plots of manually gated CD4 T cells (upper panels) or CD8 T cells (lower panels) show percentages of M_37–47_-tetramer^+^ CD4 T cells or N_339–347_-tetramer^+^ CD8 T cells (respectively marked orange or blue at 14 dpi). (**b**) Absolute numbers of M_37–47_-tetramer^+^ CD4 T cells (left panel) or N_339–347_-tetramer^+^ CD8 T cells (right panel). Results are shown as mean ± SEM, each data point represents one individual mouse (n = 4). (**c**) Histogram overlays show CD44 expression of total CD4 or CD8 T cells *versus* their respective tetramer^+^ populations at day 14 post-infection. M_37–47_-tetramer^+^ CD4 T cells and N_339–347_-tetramer^+^ CD8^+^ T cells, marked orange and blue, are gated as shown in (**a**). Data are normalized to and depicted as the percentage of the maximum count (% of max on the Y axis). Data are representative of three independent experiments. For statistics (Student’s *t* test with Welch correction), PVM-infected mice were compared to non-infected controls (day 0) as indicated.

To characterize the dynamics of PVM-specific CD4 and CD8 T cell responses in C57BL/6 mice using our newly identified MHCII-restricted M_37–47_ epitope and the previously described MHCI-restricted N_339–347_ epitope^20^, we generated peptide-loaded MHCII and MHCI tetramers, which allowed us to track virus-specific CD4 and CD8 T cell responses *in vivo*. Following PVM infection, M_37–47_-specific CD4 T cells in the lung were detectable at low levels at day 10 pi, reached a maximum of 1.8% on average among the total CD4 T cell pool at day 14 pi and declined again by day 18 pi. The N_339–347_-specific CD8 T cells exhibited a marked infiltration from day 10 pi onwards, representing up to 19.6% on average of all CD8 T cells at the peak of disease severity, and then gradually decreased **(**Fig. 3a,b **)**. In Fig. 3B this is also illustrated as total numbers of tetramer-positive CD4 or CD8 T cells. Virus-specific CD4 and CD8 T cells displayed high expression levels of CD44 compared to the total CD4 and CD8 T cell pool, suggesting that they exhibit critical effector functions during the course of PVM infection **(**Fig. 3c **)**. Staining of the PVM-specific M_37–47_ MHCII tetramer was virus-specific as CD4 T cells isolated from the airways of Influenza virus strain X31-infected mice did not show increased tetramer staining neither at day 14 (peak PVM CD4 T cell response), nor at day 8 (marked CD8 and CD4 T cell response against Influenza^29^), even though NP_366–374_ MHCI tetramer-positive Influenza-specific CD8 T cells^30^ were clearly detectable at both time points (Supplementary Fig. S3). From these data, we conclude that the modest appearance of M_37–47_-specific CD4 T cells in the airways from day 10 pi onwards is slightly delayed compared to the marked influx of N_339–347_-specific CD8 T cells at that same time point.

## Discussion

There is growing interest in the use of the natural mouse pathogen PVM to mimic and study severe pneumovirus disease. T cells play a key role in both pneumovirus clearance and disease induction, but there is a lack of tools to study T cell responses in great detail in C57BL/6 mice, which limits the use of genetically modified mice derived from this lineage. We therefore set out to identify a PVM-specific CD4 T cell epitope in C57BL/6 mice. By means *of in silico* prediction and subsequent functional validation, we were able to reveal an IA^b^-restricted epitope, corresponding to amino acids 37-47 at the N-terminal end of the PVM matrix protein. According to published sequences, the M_37–47_ epitope PMFQTSLPKNS is completely conserved in both PVM strain J3666 and strain 15^7, 31^. In comparison, IA^b^-restricted CD4 T cell epitopes in the RSV proteome have also been mapped to the M protein (M_213–223_), as well as to the M2 (M2_27–37_) and G protein (G_168–185_)^32–35^. We demonstrated that the M_37–47_ peptide exclusively stimulated virus-specific CD4 T cells, and not CD8 T cells, and induced IFNγ production in 0.2-0.3% of total CD4 T cells in the lungs. This is equivalent to the range obtained from hRSV CD4 epitope mapping studies in C57BL/6^32^. Although we did not perform direct comparisons in the same mice, we observed that the frequencies of IFNγ^+^ CD4 T cells after M_37–47_ peptide stimulation were consistently lower than frequencies identified by MHCII tetramers loaded with the same peptide. A possible explanation is that some of the antigen-specific T cells produce cytokines other than IFNγ and TNFα or maybe T cell exhaustion occurs^36^. On the other hand, this observation might also resemble the so-called functional inactivation phenotype that has been described for CD8 T cells upon hRSV and simian virus 5 infection^17, 37–39^. It is tempting to speculate that viruses from the *Pneumoviridae* and *Paramyxoviridae* families functionally inactivate all T cell subsets, though more studies are needed to address this in detail.

In this work, MHCII binding predictions for each major PVM protein were performed by means of the IEDB analysis resource tool. However, because of the less strict binding requirements and the limited predictive value of MHCII motifs, this *in silico* approach may lead to an overall lower prediction accuracy compared to MHCI binding predictions^26, 40^. No direct correlation between the IEDB-predicted percentile ranks and the percentage of IFNγ^+^ T cells elicited by each of these peptides was observed **(**Table 1 **)**. For instance, the top hit M2-2_44–58_ peptide, as reflected by a low percentile rank of 0.65, did not elicit any response in our functional screen. In contrast, The M_33–47_ epitope only had a moderate percentile rank of 4.95 but induced solid CD4-specific T cell responses **(**Fig. 2b **)**. As we have tested only 24 peptides (the 2 highest-ranked non-overlapping peptides for each of the 12 viral proteins) it is possible that the lower-ranked IEDB-predicted epitopes may harbor additional CD4 epitopes. Likewise, we cannot exclude that other peptide epitopes exist that are not predicted by the IEDB algorithm, as was shown for hRSV^18^.

In addition to the identification of an IA^b^-restricted epitope, we also performed a comprehensive kinetic analysis for both CD4 and CD8 T cell responses against PVM strain J3666 in C57BL/6 mice, which to the best of our knowledge was lacking at the time. We showed that acute PVM infection in C57BL/6 mice is associated with a large influx of activated CD4 and CD8 T cells in the lung and BAL from day 10 pi onwards, coinciding with the phase of virus elimination **(**Fig. 1a and Supplementary Fig. S1 **)**. Another study in C57BL/6 mice describes the appearance of total CD3 T cells in the alveolar space as soon as day 8 pi^13^. However, it should be noted that PVM strain 15 was used, which might explain the slight difference in kinetics. Also, in the BALB/c model, van Helden and colleagues describe a marked influx of CD8 T cells from day 10 onwards following PVM J3666 infection, similar to what we observe in C57BL/6 mice^16^. Together, these data suggest that the PVM virus induced a strong antiviral T cell response and as such might indeed play a dual role in both viral clearance and PVM-induced immunopathology as was demonstrated by others^13, 16^. Using the newly identified MHCII-restricted M_37–47_ epitope and the previously described MHCI-restricted N_339–347_ epitope, we generated fluorochrome-conjugated MHCII and MHCI tetramers, allowing us to track PVM-specific CD4 and CD8 T cells, respectively. We showed that the appearance of M_37–47_-specific CD4 T cells in the airways was slightly delayed compared to N_339–347_-specific CD8 T cells, though we currently do not know if this is a general feature for all PVM-derived epitope-specific CD4 *versus* CD8 T cell responses.

To conclude, this is the first study that demonstrates a detailed kinetic analysis of both CD4 and CD8 T cell responses against PVM strain J3666 in infected C57BL/6 mice. Moreover, the M_37–47_ MHCII-restricted epitope identified in this work, provided the basis for the development of fluorescently-labeled MHCII-peptide tetramers that serve as valuable tools to track PVM-specific T cells during the course of infection. Such tools will facilitate more detailed investigations of T-cell mediated immune responses to PVM in a variety of genetically modified C57BL/6 mice. In this respect, the use of the PVM model in general holds great promise to improve our understanding of pneumovirus-associated disease and may assist in the development of peptide-based vaccines and other novel prevention strategies.

## Methods

### Mice, virus stock and infection

Mouse-passaged stocks of PVM strain J3666 were grown as described^23^. Age-matched 7-9 week old female C57BL/6 mice were purchased from Janvier (Saint-Berthevin, France), anesthetized with isoflurane (2 l/min, 2-3%) and then infected intratracheally with a previously *in vivo* titrated sub-lethal dose of PVM in 80 µl PBS. Infections were performed intratracheally instead of the standard intranasally (i.n.) procedure in order to minimize variations. We did not observe any differences in disease severity (e.g. weight loss) between i.t. or i.n. instillations. For Influenza experiments, mice were infected i.n. with 10^3^ TCID50 H3N2 Influenza strain X31 virus in a total volume of 50 µl PBS. All experimental procedures were in accordance with institutional guidelines for animal care of the VIB site Ghent – Ghent University, Faculty of Sciences and were approved under accreditation n° EC2013-035 and EC2015-016.

### Tissue sampling and processing

Mice were sacrificed at time points indicated by intraperitoneal (i.p.) injection of sodium pentobarbital. To detect PVM-specific antibodies in the serum, blood was collected in a regular Eppendorf tube, centrifuged at 500 xg for 10 min at room temperature (RT) and the supernatant was stored at −20 °C. Bronchoalveolar lavage (BAL) fluid was collected by three subsequent injections of 1 ml PBS containing 1 mM EDTA via a tracheal catheter. Before isolation, lungs were perfused with 10 ml PBS through the right heart ventricle. The lungs were then mechanically disrupted by GentleMACS dissociation (Miltenyi Biotec) (Lung program 01_01) in RPMI 1640 (Gibco) containing 20 µg/ml Liberase and 10 U/ml Dnase (Roche), followed by digestion for 30 min at 37 °C and final GentleMacs homogenization (Lung program 02_01). Next, the cell suspension was passed through a 100 µm filter and red blood cells were lysed using ammonium chloride lysis buffer (10 mM KHCO3, 155 mM NH4Cl, 0,1 mM EDTA in MilliQ water). For RNA isolation, the lower left lung lobe was collected in 1 ml TriPure (Roche) and stored at −80 °C. The mediastinal lymph node (MLN) was sterile-smashed over a 70 µm filter in PBS and collected in a final volume of 250 µl PBS. Cell counts were performed either manually by microscopy using trypan blue and a Bürker Türk counting chamber (Marienfeld, Germany) or automatically by adding 20.000 BD Calibrite beads (BD Biosciences) to the cell pellet and subsequent flow cytometry analysis.

### Epitope prediction and peptides

MHCII binding predictions were performed on 9/25/2013 using the IEDB analysis resource Consensus tool^41, 42^. The amino acid sequences of all 12 PVM J3666 viral proteins (Genbank RefSeq accession numbers YP_173324.1-YP_173335.1)^31^ were screened for predicted epitopes in the H2-IA^b^ alleles using the default IEDB recommended prediction method. For every PVM protein, the 2 highest-ranked non-overlapping peptides were selected and synthesized. A summary of the 24 selected epitopes and their sequences is provided in Table 1. In addition to the predicted peptides shown in Table 1, the following peptides were used: M_33–43_, TVWIPMFQTSL; M_37–47_, PMFQTSLPKNS; Derp1_128–149_, GCGSCWAFSGVAATESAYLAYR^27^; N_339–347_, GAPRNRELF^20^. The M_33–43_ and M_37–47_ overlapping epitopes within the M_33–47_ peptide sequence were predicted using another IA^b^ peptide algorithm^28^. All peptides were synthesized by GenScript (Piscataway, USA), using standard chemical peptide synthesis service. The purity varied between 75% and 90%, however, for the M_33–43_, M_37–47_ and M_33–47_ peptides it was around 90%. M_33–43_ was dissolved in 10% DMSO, whereas M_37–47_ and M_33–47_ were dissolved into ultrapure water.

### Cell culture reagents and restimulation

For peptide restimulation experiments, BAL and lung single-cell suspensions (day 14 post-infection) were resuspended in sterile tissue culture medium (TCM) supplemented with 5,7% FCS (Bodinco), 1.1 mg/ml β-Mercaptoethanol (Sigma), 56 μg/ml Gentamicin (Gibco) and 1% L-Glutamine (Gibco). To assess intracellular IFNγ production, 4 × 10^5^ BAL and 2 × 10^6^ lung cells were restimulated for 6 h at 37 °C in 100 µl TCM containing 10 µg/ml peptide and 0.07% Golgistop (BD Pharmingen) in round-bottom 96-well plates. For control conditions, 10 µg/ml Derp1_128–149_ peptide or 2 µg/ml N_339–347_ peptide were added. Three biological replicates were performed, for which 10 mice per replicate were pooled to obtain sufficient cell numbers.

### Flow cytometry

Fluorochrome-conjugated antibodies were purchased from eBioscience (Temse, Belgium) [anti-CD8 eFluor450 (53-6.7), anti-CD45 APC (30-F11), anti-CD3 PE (145-2C11) and AF700 (17A2), anti-CD44 PeCy7 (IM7), anti-IFNγ PeCy7 (XMG1.2)]; BD Biosciences (Erembodegem, Belgium) [anti-CD4 BV605 (RM4-5) and anti-TNFα FITC (MP6-XT22)]; Tonbo Biosciences (San Diego, California) [anti-CD44 Redfluor710 (IM7)]. Viable cells were discriminated by the use of Fixable Viability dye eFluor780 (eBioscience). Cell surface markers were stained for 20 min at 4 °C. For peptide restimulation experiments, extracellular staining was followed by fixation and permeabilization using the CytoFix/CytoPerm solution and Perm/Wash buffer (BD Biosciences) according to the manufacturer’s protocol and intracellular IFNγ and TNFα staining was performed for 30 min at 4 °C. PE-conjugated MHCI-N_339–347_ (GAPRNRELF) and MHCI-NP_366–374_ (ASNENMETM) tetramers were purchased from Sanquin (Amsterdam, The Netherlands). PE-labeled MHCII-M_37–47_ (PMFQTSLPKNS) tetramers were generated as described previously^43^. For tetramer staining, cells were incubated with MHCI (1:15 dilution) or MHCII (10 nM) tetramer for 1 h at RT and then stained for surface markers for 20 min at 4 °C. Cells were acquired on a Fortessa cytometer equipped with FACSDiva software (BD Biosciences). Final analysis and graphical output were performed using FlowJo software (LLC, Ashland, OR).

### Viral load and PVM-specific ELISA

For viral load quantification by qRT-PCR, lungs were frozen lungs in TriPure, thawed and subsequently homogenized using the Tissuelyser II (Qiagen, Hilden, Germany). Total lung RNA was purified using TriPure isolation reagent (Roche Diagnostics) and converted to cDNA (iScript Advanced cDNA synthesis kit; Bio-Rad Laboratories), according to the manufacturer’s instructions. Quantification of PVM virus SH gene RNA and the L27 housekeeping gene was performed using the Maxima Probe qPCR Master Mix (Fermentas), hydrolysis probes (Universal probe library, Roche) and primers (IBD)^44^. Primer and probe sets were as follows: L27 set, Universal probe library #3, Fw primer TGAAAGGTTAGCGGAAGTGC, Rev primer CATGAACTTGCCCATCTCG; PVM_SH_ set, Universal probe library #66, Fw primer CACCAGATCACCCTCGAGAT, Rev primer GGGCTGGTGTAGTGTATGTGC, and results were also validated using a second PVM_SH_ set, Universal probe library #22, Fw primer AATGCACAGTTCATCCCAATC, Rev primer TTACCCGGCAGACCAGTTAC. Reactions were performed in a LightCycler 480 (Roche) at 95 °C for 10 min, 45 cycles of 95 °C for 10 s, 60 °C for 30 s, 72 °C for 1 min and a final cycle of 40 °C for 30 s. Data were analyzed using the qBase software (Biogazelle, Belgium) and PVM_SH_ expression levels were normalized to L27. PVM-specific IgG were measured in serum using the SMART M12 kit (Biotech Trading Partners, Encinitas, California), following the manufacturer’s instructions. Read-out was performed by spectrophotometry and data are represented as the absorbance index relative to a positive control.

### Statistical analysis

All experiments were performed using three to five animals per group, unless mentioned otherwise. Statistical analyses were performed using the two-tailed Student’s *t* test for unpaired data (with Welch correction assuming unequal variances) or one-way ANOVA (with Dunnett correction for multiple comparisons), making use of Prism version 7.01 (GraphPad Software, La Jolla, CA). Error bars represent standard error of the mean (SEM). Levels of significance are expressed as p-values (ns, not significant; *P < 0.05; **P < 0.01; ***P < 0.001; ****P < 0.0001).

### Data availability

All data generated or analysed during this study are included in the published article (or its Supplementary files).

## Electronic supplementary material

Supplementary Figures and legends
