# Supplementary material for: Epitope mapping and kinetics of CD4 T cell immunity to pneumonia virus of mice in the C57BL/6 strain
Source: Sci Rep. 2017 Jun 14;7:3472. doi: 10.1038/s41598-017-03042-y (PMC5471230; doi:10.1038/s41598-017-03042-y)
Supplement: Supplementary file 1 — Supplementary Figures and legends [file 41598_2017_3042_MOESM1_ESM.pdf]

Supplementary information

Epitope mapping and kinetics of CD4 T cell immunity to pneumonia virus of mice in the C57BL/6 strain

Lana Vandersarren, Cedric Bosteels, Manon Vanheerswynghels, James J. Moon, Andrew J. Easton, Gert Van Isterdael, Sophie Janssens, Bart N. Lambrecht, Mary J. van Helden

Inventory

Supplementary Figure S1, related to Figure 1
Supplementary Figure S2, related to Figure 2
Supplementary Figure S3, related to Figure 3

Figure legends

Supplementary Figure S1 | Gating strategy and dynamics of CD4 and CD8 T cell responses in PVM-infected mice. Mice were infected i.t. with a sub-lethal dose of PVM strain J3666 and sacrificed at the indicated days post-infection. (a) Representative FACS plots show gating strategy on lung cells at day 14 pi to identify CD4 and CD8 T cells by means of flow cytometry. A similar gating strategy was used to analyze MLN and BAL (data not shown). (b) Absolute numbers of CD4 (orange) and CD8 (blue) T cells in the BAL, as determined by flow cytometry. Data points are shown as mean ± SEM (n=5 per time point). (c, left panel) Representative gating strategy on lung CD4 and CD8 T cells 14 days pi to determine the frequency of CD44+ T cells (n=5 per time point). (c, right panel) Histogram overlays depict MFI of CD44 expression for both CD4 and CD8 T cells of one representative mouse at the indicated days post-infection. All results are representative of three independent experiments. BAL, bronchoalveolar lavage; MFI, mean fluorescence intensity; PI, post-infection; D, day.

Supplementary Figure S2 | Evaluation of T cell-mediated cytokine production in response to predicted MHCII-restricted PVM peptides. 8 week old C57BL/6 females were infected i.t. with a sub-lethal dose of PVM strain J3666 and sacrificed 14 days later. BAL and lung single-cell suspensions were restimulated for 6 h with each of the predicted MHCII-restricted PVM peptides (enlisted in Table 1), in the presence of Golgistop. T cells were evaluated for cytokine production by intracellular staining and flow cytometry analysis. (a) TNFα production by CD4 T cells in BAL and Lung following peptide restimulation, depicted as frequency of TNFα-producing cells among total CD4 T cells. (b) CD8 T cell-mediated IFNγ production in BAL and lung after peptide restimulation. Data are shown as frequency of IFNγ-producing cells among total CD8 T cells. As controls, cells were incubated without peptide, with an irrelevant Derp1 CD4 peptide or with a MHCI-restricted PVM $N_{339-347}$ peptide, as shown in gray. (c, left panel) Representative FACS plots (gated on CD4 T cells) show the percentage of $TNF\alpha^{+}$ CD4 T cells in response to restimulation with or without M_{33-47} peptide. (c, right panel) Histogram overlays depict CD44 expression levels of total CD4 T cells and gated $TNF\alpha^{+}$ CD4 T cell populations (marked orange in left panel) following restimulation with M_{33-47} peptide. Data are normalized to and depicted as the percentage of the maximum count (% of max on the Y axis). Results are shown as mean ± SEM from 3 biological replicates. For each biological replicate 10 mice were pooled to obtain sufficient cell numbers for epitope screening. Data are representative of two independent experiments. For statistics (Student's *t* test with Welch correction in (a) or ANOVA for multiple comparisons in (b)), conditions restimulated with peptide were compared to the no-peptide control as indicated. BAL, bronchoalveolar lavage; IFNγ, interferon gamma; TNFα, tumor necrosis factor alpha; MHCI/II, major histocompatibility complex class I or II; Derp1, *Dermatophagoides pteronyssinus* peptidase 1; Irr, irrelevant.

Supplementary Figure S3 | *In vivo* validation of the specificity of the M_{37-47} MHCII tetramer in naïve mice and PVM- or Influenza X31-infected mice. 11 week old C57BL/6 females were infected i.t. with a sub-lethal dose of PVM strain J3666 or i.n. with 10^3 TCID50 of Influenza virus strain X31. At the indicated timepoints post-infection (pi), PVM-specific CD4 T cells and Influenza X31-specific CD8 T cells in the lung were identified by flow cytometry, using M_{37-47} and $NP_{366-374}$ peptide-loaded MHCII and MHCI tetramers. (a) Representative FACS plots of manually gated CD4 T cells show percentages of M_{37-47}-tetramer^{+} CD4 T cells from PVM-infected (upper panels) or Influenza X31-infected mice (lower panels). Naïve uninfected mice were also included as a control. (b) Same data as in a, summarized in a graph with each datapoint representing one individual mouse. Results are shown as mean ± SEM (n=4). (c) Representative FACS plots of manually gated CD8 T cells show percentages of $NP_{366-374}$-tetramer^{+} CD8 T cells from naïve mice or Influenza virus X31-infected mice sacrificed at day 8 or day 14 pi. The data shown here were performed one time. For statistics, PVM-infected mice were compared to non-infected controls as indicated (ANOVA for multiple comparisons).

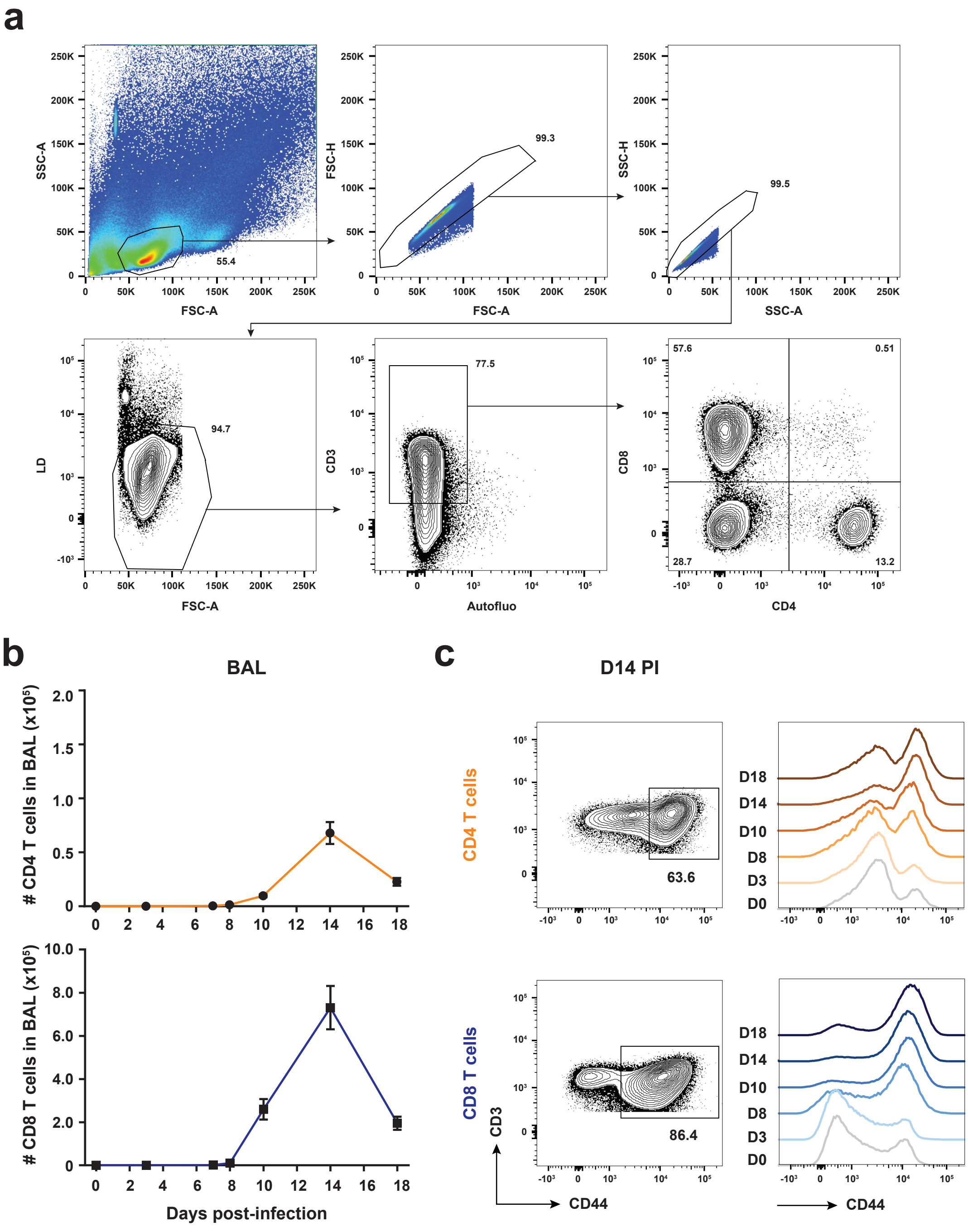

Supplementary Figure S1 | Gating strategy and dynamics of CD4 and CD8 T cell responses in PVM-infected mice.

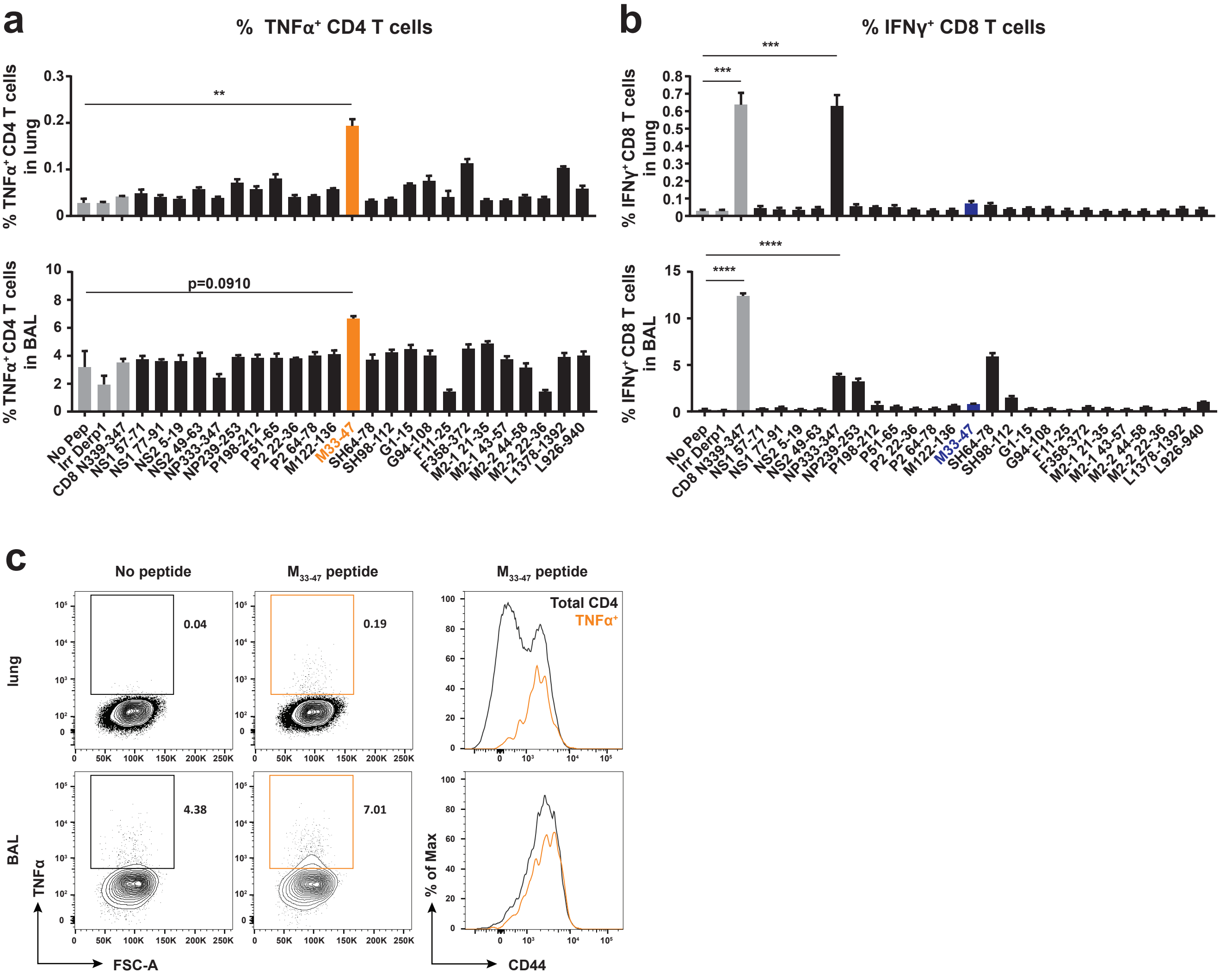

Supplementary Figure S2 | Evaluation of T cell-mediated cytokine production in response to predicted MHCII-restricted PVM peptides.

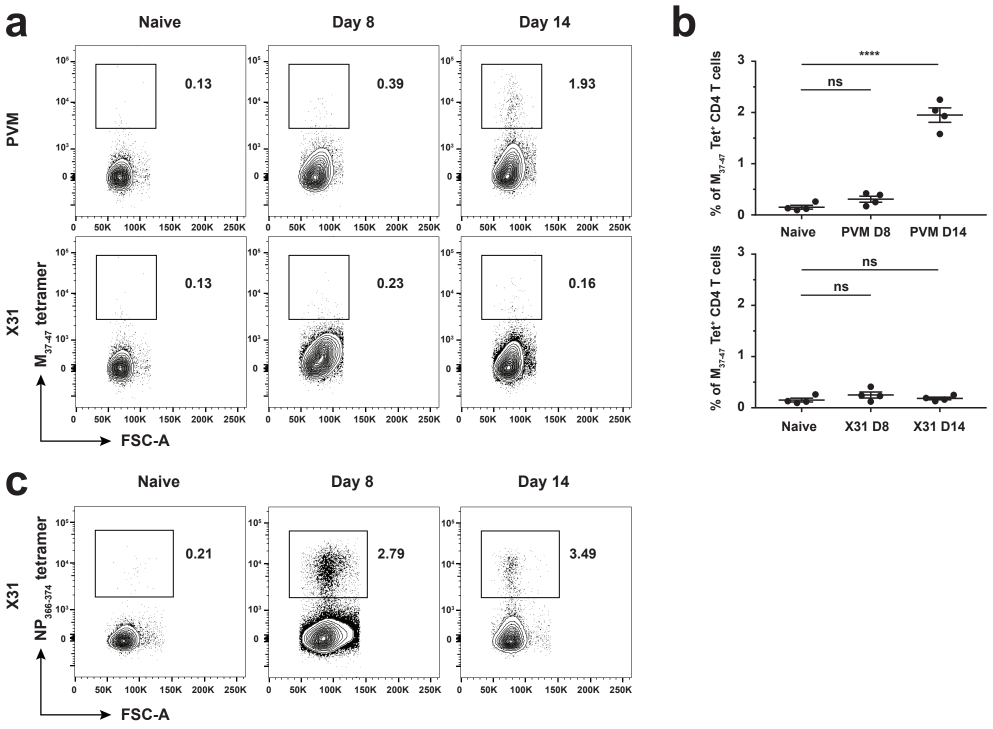

Supplementary Figure S3 | *In vivo* validation of the specificity of the $M_{37\text{-}47}$MHCII tetramer in naive mice and PVM- or Influenza X31-infected mice.
